# Distinguishing Parkinson's disease from atypical parkinsonian syndromes using PET data and a computer system based on support vector machines and Bayesian networks

**DOI:** 10.3389/fncom.2015.00137

**Published:** 2015-11-05

**Authors:** Fermín Segovia, Ignacio A. Illán, Juan M. Górriz, Javier Ramírez, Axel Rominger, Johannes Levin

**Affiliations:** ^1^Department of Signal Theory, Networking and Communications, University of GranadaGranada, Spain; ^2^Department of Nuclear Medicine, Ludwig Maximilian University of MunichMunich, Germany; ^3^Department of Neurology, University of MunichMunich, Germany

**Keywords:** Bayesian network, support vector machine, 18F-DMFP PET, Parkinson's disease, multivariate analysis

## Abstract

Differentiating between Parkinson's disease (PD) and atypical parkinsonian syndromes (APS) is still a challenge, specially at early stages when the patients show similar symptoms. During last years, several computer systems have been proposed in order to improve the diagnosis of PD, but their accuracy is still limited. In this work we demonstrate a full automatic computer system to assist the diagnosis of PD using ^18^F-DMFP PET data. First, a few regions of interest are selected by means of a two-sample *t*-test. The accuracy of the selected regions to separate PD from APS patients is then computed using a support vector machine classifier. The accuracy values are finally used to train a Bayesian network that can be used to predict the class of new unseen data. This methodology was evaluated using a database with 87 neuroimages, achieving accuracy rates over 78%. A fair comparison with other similar approaches is also provided.

## 1. Introduction

One of the neuropathological hallmarks of Parkinson's disease (PD; Greenberg et al., 2012) is a substantial decrease in the dopamine content of the striatum due to the progressive death of dopaminergic neurons of the nigrostriatal pathway (a neural pathway that connects the substantia nigra with the striatum). These marks are detectable using neuroimaging techniques, which have become an ordinary practice in the diagnosis of neurodegenerative disorders as PD. The deficiency of striatal dopamine can be assessed using a variety of nuclear medicine techniques targeted to dopamine transporters (DaT). For example, the ^123^I-ioupane (also known by its tradename DaTSCAN) is a widely-used radioligand that binds to the dopamine transporters in the striatum and allows visualizing the presynaptic striatal dopamine deciency state with high sensitivity. This drug has been successfully used to differentiate PD from healthy controls (Booij et al., 1998; Winogrodzka et al., 2003; Towey et al., 2011; Illán et al., 2012; Segovia et al., 2012). However, the effectiveness of DaTSCAN to distinguish PD from atypical parkinsonian syndromes (APS), such as multiple system atrophy (MSA; Wenning et al., 2013) and progressive supranuclear palsy (PSP; Williams and Lees, 2009), is limited due to DaT is reduced in both cases. Alternative radioligands have been proposed for this problem. For example, ^18^F-Fludeoxyglucose (^18^F-FDG) PET data, which allow evaluating the glucose metabolism of the brain, were successfully used to discriminate APS from PD in Garraux et al. (2013) and Ghaemi et al. (2002). A postsynaptic striatal deficiency can be also demonstrated by means of specific radioligands that focus on *D*_2∕3_ striatal dopamine receptors. ^123^I-Iodobenzamide (^123^I-IBZM) SPECT, ^11^C-Raclopride PET and ^18^F-Desmethoxyfallypride (^18^F-DMFP) PET have suggested for this purpose (Cordes et al., 1991; Antonini et al., 1997; Stark et al., 2007; Vernaleken et al., 2007). In addition, the latter was recently used to separate idiopathic and non-idiopathic parkinsonian syndromes with high accuracy (la Fougère et al., 2010).

Nowadays, the visual examination of the neuroimages is combined with modern computer systems that automatically analyze the data and are able to estimate their class (pathological or not). In the case of PD diagnosis, the classical approach addresses the problem directly, i.e., by quantifying the loss of striatal dopamine neurons (Antonini et al., 1997; Constantinescu et al., 2011; Garraux et al., 2013). However, modern computer systems examine the images, looking for the patterns that characterize the studied disease. Two approaches have been proposed: univariate and multivariate. On the one hand, univariate methods analyze each voxel independently, without considering the interactions between voxels. The most relevant univariate analysis approach to date is the widely used Statistical Parametric Mapping (SPM) (Friston et al., 2007). This approach has been used in a number of neuroimaging studies, including the diagnosis of neurological disorders (Hosaka et al., 2002; Wang et al., 2007; Perani et al., 2014). On the other hand, multivariate approaches consider all the voxel as a whole, evaluating the correlation of activation across brain regions (Habeck, 2010; Schrouff et al., 2013). A substantial part of multivariate methods are based on machine learning algorithms such as decision trees (Quinlan, 1986) or support vector machine (SVM; Vapnik, 1999). They have been successfully used to assist the diagnosis of Parkinsonism using different neuroimage modalities (Cordes et al., 1991; Dodel et al., 2003; Illán et al., 2012; Segovia et al., 2012; Nair et al., 2013; Mudali et al., 2015). Bayesian approaches (Ben-Gal, 2008) were also used for this purpose. In Towey et al. (2011), a naive Bayes classifier is used along with Principal Component Analysis in order to separate parkinsonian and non-parkinsonian syndromes. Several Bayesian methods were also evaluated in Morales et al. (2013), achieving accuracy rates over 90% when predicting dementia development in PD patients.

In this work, we demonstrate a method based on SVM classification and Bayesian networks to separate idiopathic PD from APS using ^18^F-DMFP PET data. Initially we performed a selection of the regions of interest (ROIs) for this problem using a *t*-test. The selected regions were then evaluated by means of a SVM classifier. Subsequently, a Bayesian network was developed using the outputs of the SVM classification procedure. Finally, the Bayesian network was used to classify new unseen data. This method was evaluated using a database with 87 DMFP neuroimages labeled as idiopathic PD, MSA or PSP. Accuracy rates over 78% were obtained when separating PD from APS (MSA or PSP), outperforming other previous approaches.

## 2. Materials and methods

### 2.1. Neuroimaging data

Neuroimaging data used in this work was collected in a longitudinal study carried out in the University of Munich (la Fougère et al., 2010). Eighty-seven (87) patients with parkinsonism, previously confirmed by a ^123^I-FP-CIT SPECT scan according to widely accepted criteria (Koch et al., 2005), were undergone *D*_2∕3_ receptor imaging with ^18^F-DMFP. Sixty (60) min after the radiopharmaceutical injection the neuroimaging data were acquired by means of a ECAT EXACT HR^+^ PET scanner (Siemens/CTI). The emission recording consisted of 3 frames of 10 min each, acquired in 3-dimensional mode. The resulting images were reconstructed as 128 × 128 matrices of 2 × 2 mm voxels by filtered backprojection using a Hann filter with a cutoff frequency of 0.5 Nyquist and corrected for randoms, dead time, and scatter.

The patients were clinically monitored during the following years. Two years after the data acquisition, the neuroimages were labeled by experienced clinicians on the basis of last observations. According to the United Kingdom Parkinson Disease Society Brain Bank Diagnostic Criteria for Parkinson Disease (Hughes et al., 2002), the second consensus statement on the diagnosis of multiple-system atrophy (Gilman et al., 2008) and the established criteria for the diagnosis of progressive supranuclear palsy (Litvan et al., 1996), 3 groups were defined: idiopathic PD, MSA, and PSP patients. Demographic details of the groups are shown in Table 1.

**Table 1:** **Sex and age information of the groups of patients considered in this work**.

	**#**	**Sex**		**Age**		
		**M**	**F**	**μ**	**σ**	**Range**
Idiopathic PD	39	22	17	61.38	11.14	35-81
MSA	24	20	4	68.42	10.73	43-85
PSP	24	12	12	69.29	7.33	55-84

μ and σ stand for the average and the standard deviation respectively.

In order to ensure that any given voxel in different images refers to the same anatomical position across the brains, all the images were spatially normalized using the template matching approach implemented in the SPM software (version 8). An *ad-hoc* template was generated for this procedure: First, idiopathic PD neuroimages were registered to a randomly chosen one. The resulting images and their hemisphere midplane reflections were then averaged, obtaining a symmetric image. That way, effects due patients affected by non-bilateral PD variants are minimized (Djaldetti et al., 2006). Finally, this image was smoothed and used as template.

After the spatial normalization, the intensity of the images was also normalized to a value *I*_*max*_, obtained by averaging the 0.1% of the highest intensities per image, as described in Saxena et al. (1998).

### 2.2. Background on bayesian networks

Bayesian networks (a.k.a. belief networks) are statistical models belonging to the family of probabilistic graphical models that combine principles from graph theory, probability theory, computer science, and statistics (Ben-Gal, 2008). In particular, they are directed acyclic graphs (DAG) in which nodes represent random variables in the Bayesian sense (observable quantities, latent variables, unknown parameters or hypotheses) and edges between the nodes represent probabilistic dependencies among the corresponding random variables. A Bayesian network defines a unique joint probability distribution given by:

(1) $$\begin{array}{l} p\left(x^{\left(1\right)},\ldots ,x^{\left(M\right)}|y\right)=\overset{M}{\underset{m=1}{\prod }}p\left(x^{\left(m\right)}|Pa_{x^{\left(m\right)}},y\right)=\overset{M}{\underset{m=1}{\prod }}\theta_{x^{\left(m\right)}|Pa_{x^{\left(m\right)}}} \end{array}$$

where **x** = {*x*^(1)^, …, *x*^(*M*)^} and *y* respectively denote a set of random variables and a property of them. In our case **x** represents the neuroimaging features for a given patient and *y* is the label assigned to that patient. θ is the set of parameters that quantifies the conditional probability distribution in the network and $Pa_{x^{\left(m\right)}}$ represents the set of parents of *x*^(*m*)^ in the graph.

When the topology and/or the parameters of the network are unknown, they can be estimated using a set of training data. Two approaches are commonly used to learn the network structure: constraint-based and search-and-score. The former starts with a fully connected graph, and remove edges if certain conditions are satisfied in the training data. The latter approach performs an exhaustive search in the space of all possible structures, which are evaluated using a predefined scoring function.

Once the structure is learned and the parameters are fixed, the network can be used for inference. In this case, the Bayesian network encodes a disruption *p*(*x*^(1)^, …, *x*^(*M*)^, *y*) so that, given a set of features, *x*^(1)^, …, *x*^(*M*)^, it returns the label *y* that maximizes the posterior probability *P*(*y*|*x*^(1)^, …, *x*^(*M*)^), which is trivially derived from Equation 1 using the definition of conditional probability and the chain rule.

### 2.3. Background on support vector machines

Support vector machine is a supervised learning method derived from the statistical learning theory, which was developed by Vladimir Vapnik in late 90s (Vapnik, 1999). A SVM classifier builds a function *f* : ℝ^*D*^ → {±1} using the training data (*n D*-dimensional patterns, {**x**_1_…, **x**_*n*_}, and their class labels, {*y*_1_, …, *y*_*n*_}) so that *f* is able to predict the label *y*_*i*_ of a new example **x**_*i*_.

SVM can use kernelized inputs that allow us learning a nonlinear function or decision boundary. More sophisticated variants based on multiple kernel learning (MKL) define the kernel function as the combination of other simpler kernels (Gonen and Alpaydin, 2011):

(2) $$\begin{array}{l} k\left({\text{x}}_{i}{\text{x}}_{j}\right)=f\left({\left\{k_{p}\left({\text{x}}_{i}^{p},{\text{x}}_{j}^{p}\right)\right\}}_{p=1}^{P}\right) \end{array}$$

where kernel *k* is computed as the combination of *P* kernels each of which taking a feature representation of data instances: ${\text{x}}_{i}={\left\{{\text{x}}_{i}^{p}\right\}}_{p=1}^{P}$ where ${\text{x}}_{i}^{p}\in {\mathbb{R}}^{D_{p}}$ and *D*_*p*_ is the dimensionality of the corresponding feature representation.

### 2.4. Selection of regions of interest

**Figure 1:**
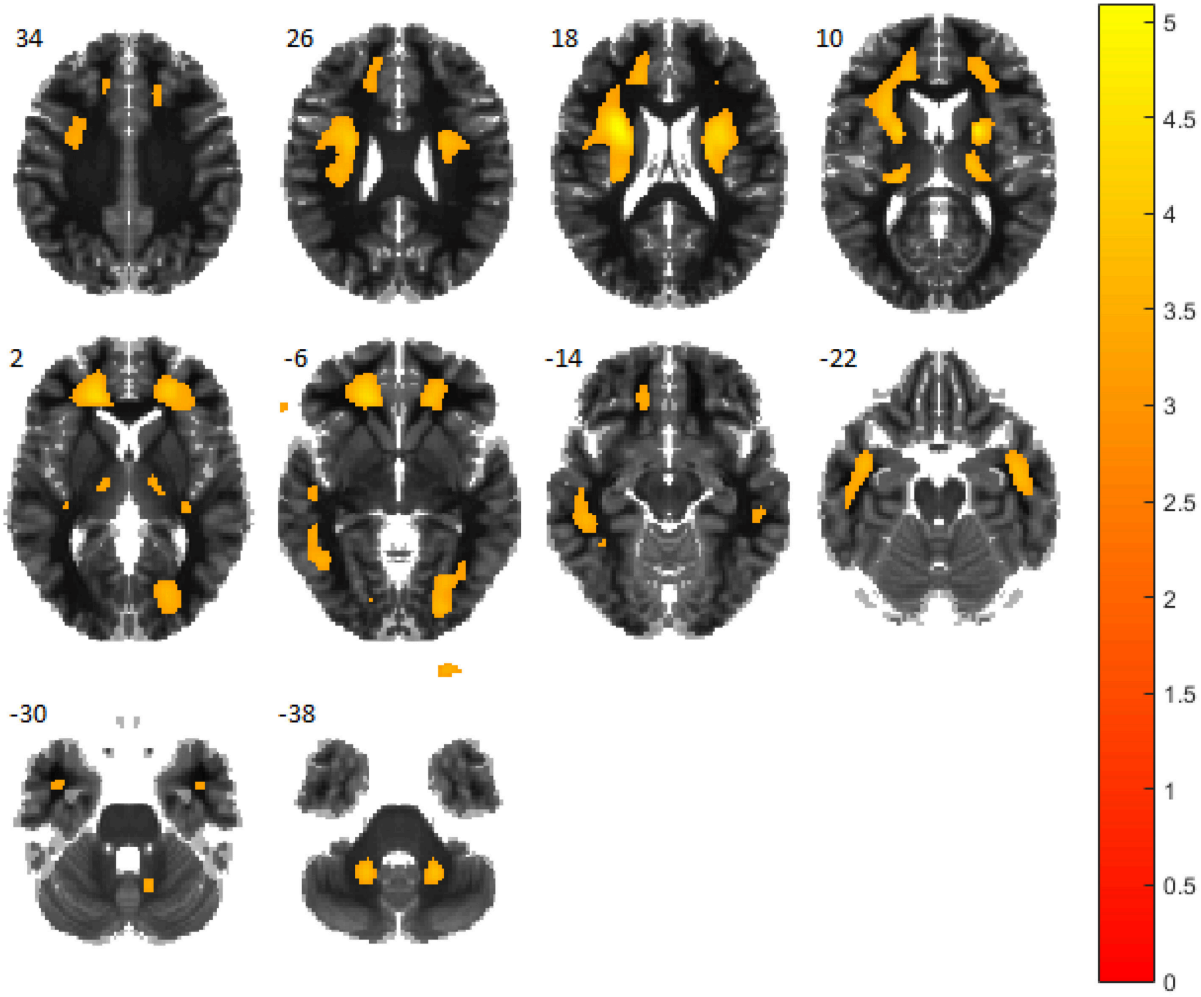
***t*-test comparing patients with idiopathic PD and atypical parkinsonian syndromes**. Regions in orange/yellow are significantly lower (*p* < 0.001, uncorrected) in idiopathic PD patients. Observe that most part of the thalamus, anterior cingulate gyrus and pars opercularis are covered by highlighted areas.

^18^F-DMFP has a high binding affinity for dopamine transporters in the striatal region of the brain. For this reason, neuroimaging studies based on it usually focuses on that region. However, ^18^F-DMFP PET neuroimages contain a substantial part of the total intensity in regions other than the striatum. In order to reveal the most important regions to separate idiopathic PD from APS we carried out an univariate analysis. Specifically we performed a two-sample *t*-test comparing both populations under the hypothesis of data corresponding to idiopathic PD patients have lower intensity than those from APS patients. The test was carried out using the SPM software (version 8) and a smoothed version of the neuroimaging data. The full-width at half maximum of the Gaussian smoothing kernel was fixed at 8 mm. The resulting map, thresholded at *p* < 0.001 (uncorrected), is shown in Figure 1.

Finally, the *t*-test map was matched with the Automated Anatomical Labeling (AAL) atlas (Tzourio-Mazoyer et al., 2002) and the regions of the atlas with the highest proportion of highlighted voxels in the *t*-test map were selected.

### 2.5. Multivariate analysis based on support vector machines and bayesian networks

Once a reduced set of regions of interest has been selected, the accuracy of each region to discriminate between groups was computed using a SVM classifier and a leave-one-out (LOO) cross-validation (CV) scheme. As a result, an accuracy value was assigned to each region.

**Figure 2:**
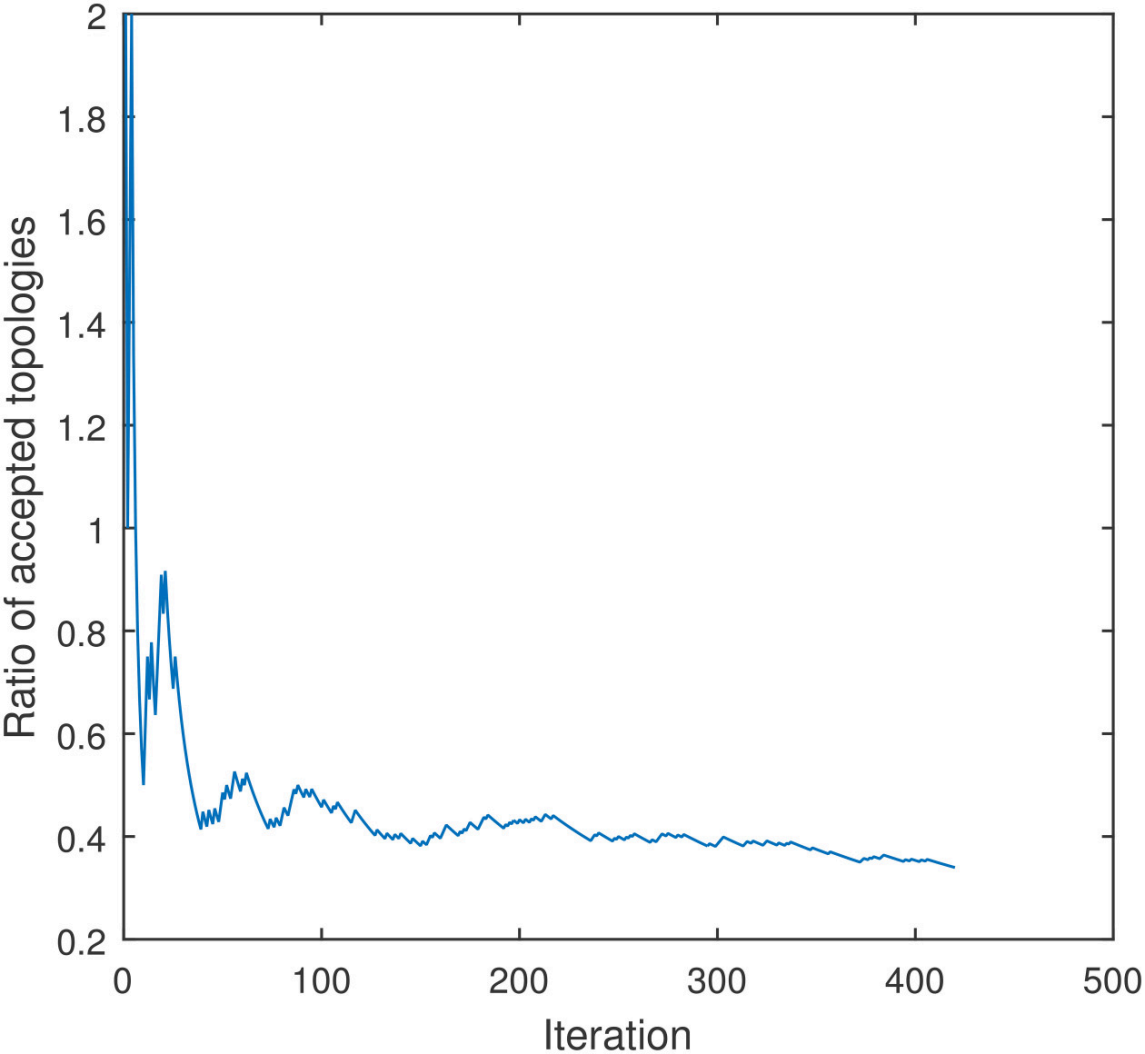
**Convergence of the ratio of accepted topologies of the Metropolis-Hasting method used to estimate the structure of a Bayesian network for a model with 4 regions**. The algorithm was trained using the accuracy of each region to separate idiopathic and non-idiopathic PD patients.

The structure of the Bayesian network is then estimated by means of a Metropolis-Hastings (MH) algorithm (Metropolis et al., 1953; Hastings, 1970), a Markov chain Monte Carlo (MCMC) method that converges after about 200 iterations (see Figure 2) and is more efficient than the more popular search-and-score approach. This algorithm was implemented to use the Bayesian information criterion as the score function to find the optimal structure (Heckerman et al., 1995).

**Figure 3:**
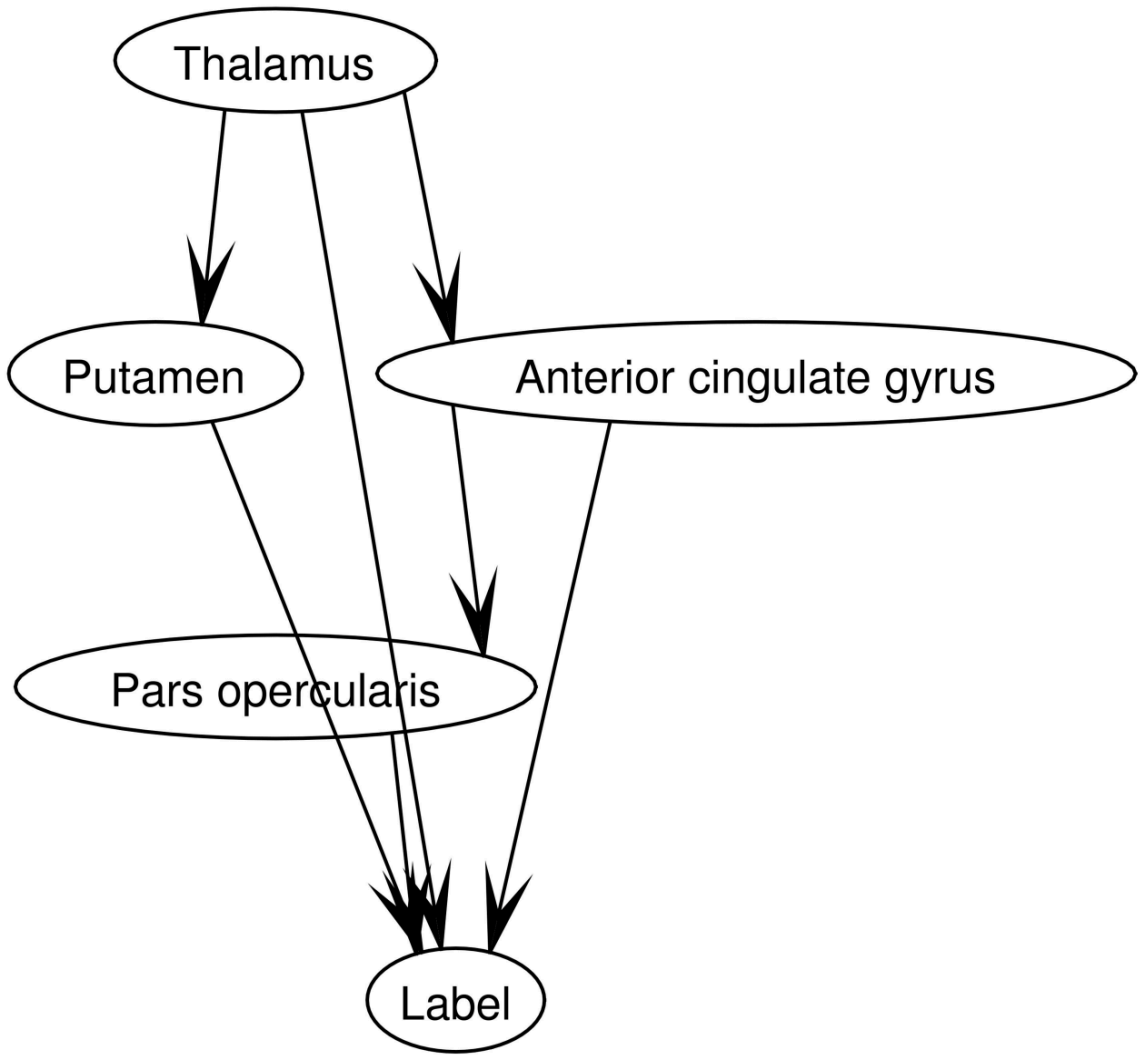
**Topology of a Bayesian network for a model with 4 regions**. They were selected using SPM as described in section 2.4. The network structure was learned using a Metropolis-Hastings algorithm.

The parameters of the model were calculated by means of a maximum a posteriori scheme, which assumes that all the variables are fully observed. The accuracy of each region previously computed was used in this procedure as training data. Figure 3 shows a Bayesian network for a model with 4 regions. Observe that the labels are included in the graph as an additional node. This allows using the network for inference (Illan et al., 2014).

Finally, the Bayesian network was used to classify new unseen data by selecting the label that maximizes the posterior probability. If the two possible labels have equal probability (0.5 in both cases), the label with the most *votes* was selected.

## 3. Experiments and results

The methodology proposed above was evaluated using the neuroimaging data described in section 2.1. In order to avoid biased results, a LOO-CV scheme was implemented. Since another LOO-CV loop was used to estimate the accuracy of the regions, this resulted in a 2 levels (nested) CV. This strategy has been suggested as an effective method to evaluate the risk of a classification procedure (Varma and Simon, 2006), especially when the database is small. The Bayes Net Toolbox (Murphy, 2001) and LIBSVM (Chang and Lin, 2011) package were respectively used for structure learning and SVM classification. They were parametrized to use the default values, including the cost parameter of the classifier, *C*, which was fixed to the commonly accepted value of 1. The SVM classifier used linear kernels. The pseudo-code that describes all the experiments is shown in Algorithm 1.

**Algorithm 1:**
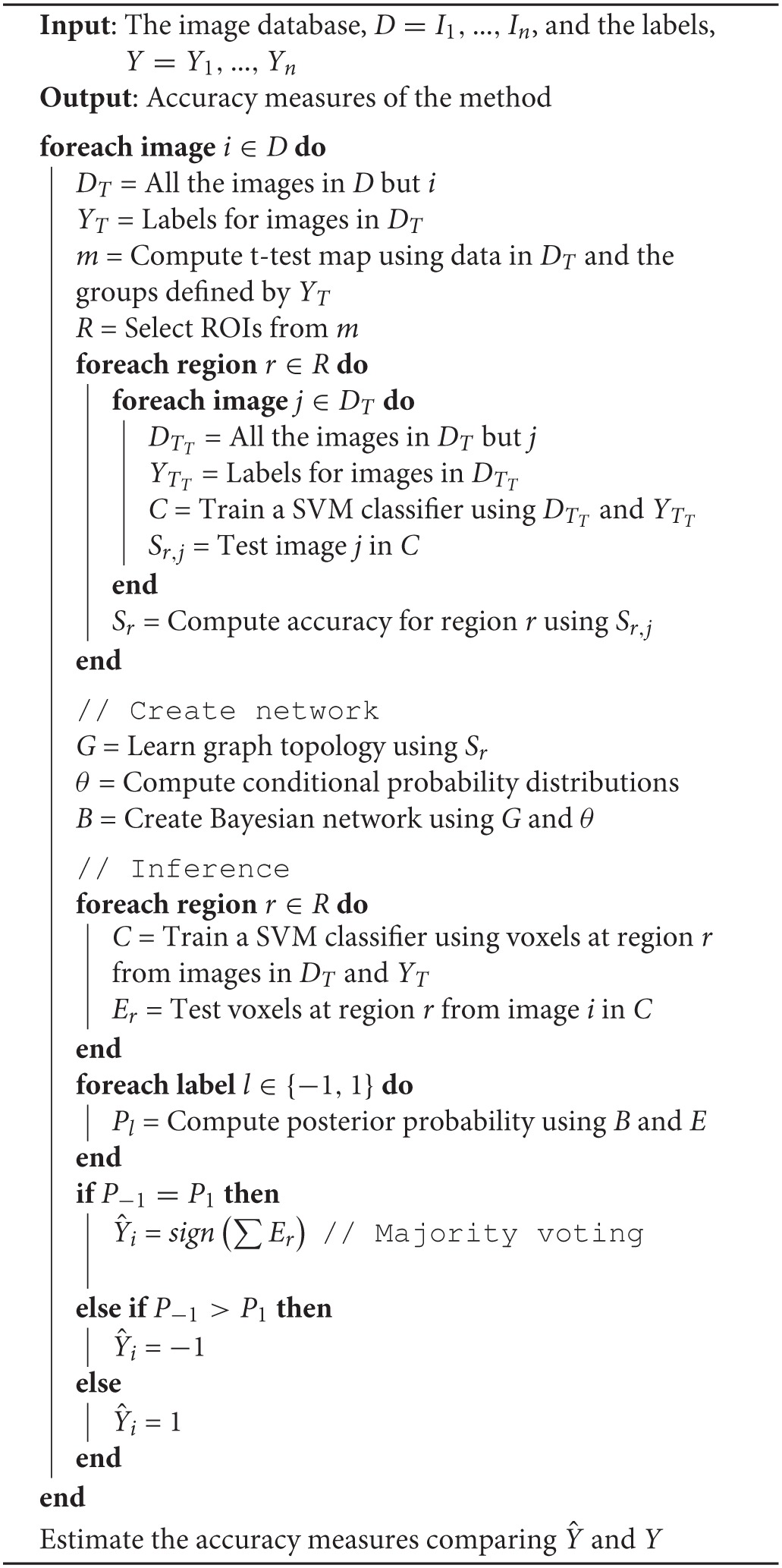
Evaluation procedure

Table 2 shows the accuracy measures obtained by the proposed method. The results are compared with those obtained by other methods based on SVM classification: (i) a *Voxel-As-Feature* approach, which consists on using all the voxels as feature, (ii) the classical approach for PD diagnosis consisting on using only voxels in the striatum area, (iii) a method that uses the voxels belonging to the selected regions as a whole, (iv) a multiple classifier approach with a classifier per selected region and a majority voting strategy, and (v) a multiple kernel learning algorithm with a kernel per region.

**Table 2:** **Classification performance of the proposed algorithm compared with other approaches**.

	**Accuracy %**	**Sensitivity %**	**Specificity %**	**Positive likelihood**	**Negative likelihood**
All the voxel in the brain	70.11	61.54	77.08	2.69	0.50
Only striatum	73.56	69.23	77.08	3.02	0.40
Selected regions as a whole	70.11	66.67	72.92	2.46	0.46
Multiple SVM (majority voting)	74.71	74.36	75.00	2.97	0.34
Multiple kernel SVM	75.86	71.79	79.17	3.45	0.36
Proposed method (Bayesian network)	78.16	76.92	79.17	3.69	0.29

## 4. Discussion and conclusions

Distinguishing between Parkinson's disease and atypical parkinsonian syndromes is still a challenge due to both disorders have similar symptoms (Litvan, 1999). Still, the methodology we demonstrated achieved an accuracy rate over 78% and a good trade-off between sensitivity and specificity. These results suggest the proposed method is suitable to assist the diagnosis of PD and confirm the usefulness of DMFP data for this purpose.

Comparison with previous works in terms of classification accuracy is difficult because of the lack of studies using DMFP data for the same purpose. The separation of PD from other non-idiopathic parkinsonian syndromes have been previously addressed using multiclass classification procedures and MRI or PET data with ^18^F-FDOPA, ^18^F-FDG or ^11^C-RACLO (Ghaemi et al., 2002; Eckert et al., 2004, 2005; Spetsieris et al., 2009; Garraux et al., 2013). Reported accuracy rates vary from 68.5% up to 90%. This value depends on the database (the number of groups, the criteria to assign the labels, etc.) and the methodology (usually a binary classification is simpler than a multiclass procedure). The methodology proposed in this work achieved an accuracy rate of 78.16% and outperformed other previous approaches in a fear comparison (using the same data and classification approach), as shown in Table 2. The relatively low accuracy rates obtained, in general, by all the methods evaluated in this work is explained by the neuroimages we used, which correspond to early stages of the disease. Observe that the neuroimaging data were acquired during the first visit, 2 years before assigning the final diagnosis that was used to label the data. Furthermore, it is worth noting that the database was clinically labeled, what introduced an error due to the intrinsic limitations of the clinical assessment (Jobst et al., 1998), and the generalization the of classification procedure should be interpreted from this perspective.

In addition to the putamen, whose relation with PD is widely accepted, other regions were identified by the univariate analysis. Namely, the thalamus, anterior cingulate gyrus and pars opercularis (a subregion of the inferior frontal gyrus) showed differences when comparing PD and APS patients (see Figure 1). These three areas has been previously reported as affected by PSP and not affected by MSA or PD what facilitates separating PSP patients from the PD group (Zgaljardic and Feigin, 2004; Varrone et al., 2007; Messina et al., 2011).

One of the main feature of the proposed method is the combination of univariate and multivariate analyses. Both approaches are widely used for diagnosis purposes, however, the former is more suitable for groups comparison while the latter can be easily used for classification. For that reason, we used a univariate analysis for the selection of the regions of interest and a multivariate analysis to separate the groups. This second analysis was based on SVM classification and Bayesian networks. The data was first analyzed, region by region, in a SVM classifier and its outputs were then managed by a Bayesian network to provide a final output. Thus, the Bayesian network can be seen as a way of weighting the SVM decisions for the individual regions. Therefore, this methodology can be used as an alternative to the majority voting strategy and other approaches to deal with multiple classifier decisions in systems for a single classification problem. The main advantage of Bayesian networks in this context is their ability to take into account the information about the relations between regions. The obtained results suggest that this information is useful to separate PD and APS patients. If reproduced by other studies, it could improve the diagnosis of these disorders.

As other computer aided diagnosis systems, the method proposed in this work could be a valuable tool for the clinical practice. Using a database properly labeled, the system is able to analyze neuroimaging data from a new patient and estimate the disorder he/she suffers. However, this procedure should be supervised by experienced clinicians to corroborate that the diagnosis estimated by the system is consistent with the patient's symptoms.

### Conflict of interest statement

The authors declare that the research was conducted in the absence of any commercial or financial relationships that could be construed as a potential conflict of interest.
